# Effect of the multicomponent healthy high school intervention on meal frequency and eating habits among high school students in Denmark: a cluster randomized controlled trial

**DOI:** 10.1186/s12966-021-01228-2

**Published:** 2022-02-04

**Authors:** Katrine Sidenius Duus, Camilla Thørring Bonnesen, Johanne Aviaja Rosing, Katrine Rich Madsen, Trine Pagh Pedersen, Mette Toftager, Lau Caspar Thygesen, Rikke Fredenslund Krølner

**Affiliations:** National Institute of Public Health, University of Southern Denmark (SDU), Studiestræde 6, 1455 Copenhagen K, Copenhagen, Denmark

**Keywords:** School-based intervention, Adolescents, Meal frequency, Eating habits, Breakfast, Lunch, Water intake, Fruit and vegetables, Students, Randomized controlled trial

## Abstract

**Background:**

Previous studies have shown that multicomponent interventions may improve meal frequency and eating habits in children, but evidence among young people is limited. This study evaluated the effect of the Healthy High School (HHS) intervention on daily intake of breakfast, lunch, water, fruit, and vegetables at 9-month follow-up.

**Methods:**

The study included first-year students (≈16 years) attending high school in Denmark. Participating schools were randomized into the HHS intervention (*N* = 15) or control group (operating as usual) (N = 15). The intervention was designed to promote well-being (primary outcome) by focusing on healthy habits including meals, stress prevention, and strong peer relations. It included a curriculum, structural and organisational initiatives, a workshop, and a smartphone application. Students completed self-administered online questionnaires at the beginning of the school year and nine months later. To account for clustering of data, we used multilevel logistic regression analyses to estimate odds ratios (OR). We applied an intention-to-treat approach with multiple imputations of missing data.

**Results:**

At baseline 4577 of 5201 students answered the questionnaire and 4512 at follow-up. In both groups the proportion of students eating breakfast decreased from approximately 50% to 40% from baseline to follow-up, and lunch frequency decreased from approximately 50% to 47%. Daily water intake, intake of fresh fruit and intake of vegetables remained unchanged from baseline to follow-up. There were no significant between group differences on any of the outcomes at first follow-up: breakfast: OR = 0.85 (95% CI: 0.65;1.10), lunch: OR = 0.96 (95% CI: 0.75;1.22), water intake: OR = 1.14 (95% CI: 0.92;1.40), intake of fresh fruit: (OR = 1.07, 95% CI: 0.84;1.37), vegetables: (OR = 1.01, 95% CI: 0.77;1.33).

**Conclusion:**

No evidence of an effect of the HHS intervention was found for any of the outcomes. Future studies are warranted to explore how health promoting interventions can be integrated in further education to support educational goals. Moreover, how to fit interventions to the lives and wishes of young people, by also including systems outside of the school setting.

**Trial registration:**

ISRCTN, ISRCTN43284296. Registered 28 April 2017 - retrospectively registered.

**Supplementary Information:**

The online version contains supplementary material available at 10.1186/s12966-021-01228-2.

## Introduction

Eating patterns among Danish high school students (≈15–20 years old) are, as among most young people in western countries, characterized by more frequent meal skipping than in younger children [1, 2] and a low intake of water [3, 4], fruit, and vegetables [1–3, 5]. Danish Authorities recommend a daily intake of at least 600 g of fruit and vegetables and approximately 1–1.5 l water per day [6]. Up to half of Danish 15–18 year-olds skips breakfast at least once a week, and two-thirds skip lunch [2, 3, 7]. Two-fifths of high school students eat fruit daily, and more than 90% eat vegetables at least 2–4 times a week [2, 7]. In Denmark, water represents on average only 44% of 13–18-year-olds’ daily fluid intake corresponding to about 700 ml/day [8]. Furthermore, girls tend to eat more fruit and vegetables than boys [1, 3, 7, 9] but also to skip breakfast more often [1, 7, 10, 11]. Children and young people of low socioeconomic position report a lower intake of fruit and vegetables [1, 9] and to skip breakfast [1, 12, 13] and lunch [12, 13] more often than children and young people of high socioeconomic position.

The eating patterns among young people are worrying as they may negatively affect diet quality [10, 14], health, and well-being [15–18] but also track into adulthood with an increased risk of non-communicable diseases [19–24]. Healthy meal and eating habits may contribute to the prevention of overweight and obesity in young people [15] as well as maintenance of a healthy body weight and optimal cardiometabolic health [25]. Moreover, regular meals positively affect cognitive function, academic performance, learning abilities and school attendance [17, 26, 27]. The replacement of juice, milk, and diet or sugar-sweetened beverages (SSB), with water may reduce the total energy intake [28] and be beneficial in weight management [29].

Internationally, evidence regarding effective school-based multicomponent interventions targeting meal frequency and eating habits among young people (> 16 years) is limited [30–32]. Similarly, no previous dietary intervention studies have targeted this older age group in Denmark, while some multicomponent intervention studies have succeeded in improving dietary outcomes among children and adolescents in primary schools [8] through free provision of school meals [33, 34] and fruit and vegetables [35].

Among children (< 16 years) international reviews suggest that school-based multicomponent interventions including educational, policy, and environmental components, as well as parent involvement, are more effective in promoting healthy meal habits than single-component interventions [30, 32, 36]. Moreover, healthy and unhealthy behaviours tend to cluster [17, 37, 38] and multi-behavioural interventions aimed at improving nutrition and physical activity simultaneously have shown a better effect on weight-related outcomes than dietary or physical interventions alone [30]. This calls for a broad approach to health promotion among young people, that consider multiple factors at the same time [38]. A needs assessment among Danish high school students and staff found that students experienced that their transition to high school had resulted in unhealthy eating habits and more frequent meal skipping which made them feel tired and unable to concentrate. Most student brought their lunch from home as shown in another study [39]. Other options were to buy food in the school canteen or nearby supermarkets and restaurants. Student expressed that the options in the canteen made them eat unhealthier and they would like to have healthier options. We also found that the canteen management most often has full autonomy of their selection primarily because they are run by private actors. Making it an interesting arena for an intervention. Interviewed principals, teachers, and student counsellors highlighted that compared to primary school high schools were characterized by more self-dependent students and less school-home collaboration with parents, which questions whether a parental component is suitable in the high school setting (Bonnesen et al. *unpublished*).

This paper aims to examine the effect of the multi-behavioural multicomponent Healthy High School (HHS) intervention on meal frequencies and eating habits among first-year high school students (≈16 years old) at the end of the school year, in which the intervention was implemented. The paper reports the findings for daily intake of breakfast, lunch, and amount of daily water intake (secondary outcomes of the HHS study), and intake of fruit and vegetables (explorative outcomes of the HHS study). Furthermore, we will examine if the intervention effect differs by gender and socioeconomic position.

## Methods

### The HHS intervention

The overall aim of the HHS intervention was to promote well-being (primary outcome) among first-year high school students in Denmark. The intervention was tested in a cluster-randomized controlled trial (RCT). The trial is registered in ISRCTN, ISRCTN43284296 (28 April 2017 - retrospectively registered) and the trial design been described in detail elsewhere [40].

The Intervention Mapping approach was used to develop the programme theory and intervention components and to plan the evaluation systematically based on a comprehensive needs assessment compiling evidence, theory, and knowledge about the context and target group [40, 41]. As part of the needs assessment we conducted 1) 16 focus group interviews with students (*n* = 74), two focus group interviews (*n* = 7) and five single interviews with principals, teachers, and student counsellors at high schools and telephone interviews with two canteen managers; 2) a brainstorm session with students and school staff to explore the feasibility of early intervention ideas and schools’ capacity to implement; 3) an epidemiological assessment using existing questionnaire data from more than 70.000 Danish high school students; 4) literature reviews of determinants of the five secondary outcomes and previous school-based multicomponent or multiple health behaviour change interventions (Bonnesen et al. *unpublished*).

The needs assessment identified stress prevention and promotion of regular meals, physical activity, and sleep as well as students’ sense of community at school as five important pathways to achieve a higher level of well-being (Bonnesen et al. *unpublished*). The intervention consisted of four intervention components (Table 1) which combined educational and environmental initiatives and were designed to change important and modifiable determinants of these five secondary outcomes: a curriculum; a catalogue specifying organisational and environmental changes for a supportive school environment; ‘Young & Active’ - a peer-led innovation workshop to initiate school-based movement activities; and a smartphone application (app) [40] (Bonnesen et al. *unpublished*). The intervention was implemented among first-year high school students in the school year 2016/2017. This paper focuses on the dietary outcomes of the intervention. The primary outcome (well-being) as well as the outcomes of the four other pathways to well-being will be reported elsewhere.

**Table 1:** The Healthy High School (HHS) intervention components

1) **The HHS curriculum**	
The curriculum (accessible in Danish here: www.sdu.dk/da/sif/bgym) is based on behaviour change techniques to change social norms and cognitive factors such as knowledge, awareness, skills, and attitudes. It consists of a total of 17 mandatory lessons (1440 min) for four different subjects (Danish, Social Studies, Physical Education and Sport and Introduction to Natural Science), and optional lessons within a Multi-Subject Coursework (one school week). Activities focusing on healthy eating habits and meal frequency are included in 5 lessons (405 min) and consist of 1) information on national dietary guidelines and training of skills to critically evaluate different sources of dietary advise, 2) sociology of food (Discussion of the importance of norms, habits, and health inequalities on food habits), 3) the interrelations between eating habits and meal frequency and stress, sleep habits, and physical activity. All lessons were designed to cover official learning goals defined by the Danish Ministry of Education and were intended to replace some of the standard lessons for the subjects mentioned above. The teachers were introduced briefly to the material at a pre-intervention kick-off conference but were expected to be able to deliver the activities without prior training.	
2) **The HHS catalogue – organisational and environmental changes for a supportive school environment**	
The catalogue (accessible in Danish here: www.sdu.dk/da/sif/bgym) consists of 16 initiatives addressed to school management, canteen staff, student councils, teachers, and student counsellors. Two initiatives aimed directly at creating a school environment that support regular meals, and intake of water and healthy snacks. **Initiative 1:** A visit from a professional canteen consultant at the beginning of the school year. The consultant encouraged the canteen staff to adjust their food selection to support regular meal frequency, healthy snacks, and intake of water. The consultant had formal training in nutrition and health and profound experience in consulting canteens. The canteen consultant used the validated scoring system “*Kantinetjekket|Buffet*” (in English “*Canteencheck|Buffet*”) [42] to rank the canteens nutritional value. Based on the result, the consultant guided the canteen staff in implementing changes that could improve the nutritional value of their selection of food and nudging strategies which could promote healthy choices. The initiative focused on changing one or a few things at a time and to substitute existing products with a healthier version, e.g., white bread and pasta with wholegrain versions. Halfway through the school year, the canteens received a mini catalogue with further guidance and ideas to make a healthier canteen (accessible in Danish here: www.sdu.dk/da/sif/bgym). This catalogue was based on the canteen consultant’s experiences from the visits at all intervention schools and developed together with the project group. **Initiative 2:** We encouraged the school management to enable access to cold, clean water from a hygienic source. Three suggestions were given: 1) installing water fountains, 2) make pitchers with cold water available daily at the school area, 3) to provide easily accessible and cheap bottle water. The catalogue also includes other initiatives to support healthy eating habits and regular meals e.g., formulation of a health- and well-being school policy and common breakfast for all first-year students. The catalogue was presented to representatives of the intervention schools at a pre-intervention kick-off conference.	
3) **Young & Active – a peer-led innovation workshop and derived activities**	
Based on the needs assessment the innovation workshop specifically aimed at inspiring students to develop and implement new activities to promote physical activity and sense of community at school and did not address healthy eating habits and meal frequencies. The workshop was based on a peer-led approach, student co-determination, innovation techniques and local solutions. At all intervention schools the workshop was facilitated during school hours by university students in Sport Science and Health (peer mentors) within the first months of the intervention. The workshop included two parts of 90 min each, held at two separate days with one or two weeks in between.	
4) **The HHS smartphone application (app)**	
The app aimed to support and promote healthy habits and well-being outside school hours. The app is designed to influence student’s skills, knowledge, awareness, outcome expectations, and attitude. The app includes e.g., 1) healthy breakfast, lunch, and snack recipes, 2) meal tracking options, 3) quizzes, tests, and debunking myths on healthy eating habits and regular meals. Furthermore, the students could sign up for an eight-week text messaging programme on how to e.g., improve eating habits. Contrary to the original implementation plan, the app was introduced to the students 12 weeks after the introduction of the intervention due to a delayed delivery from the app developing company which hindered students’ uptake of the app. The download of the app was promoted by teachers at the school and the project group and needed a code to install. (The app is no longer available for download).	

### Setting

Education in Denmark is financed by taxes and therefore free of charge. All children in Denmark are obligated to primary schooling from age 6–7 to 15–16 years (9th grade). Afterwards they may choose to attend further education (e.g., upper secondary school – *admission can require certain grades or assessments from teachers or counsellors*) or to enter the labour market without prior training. The upper secondary school leaving examination is one of five general upper secondary education and training programmes in Denmark that qualifies for access to higher education. In this paper we apply the term ‘high school’ (in Danish: *gymnasium*) to refer to this three-year program [43]. Approximately 70% of young people in Denmark get enrolled in high school, after they finish 9th or 10th grade. Thus the high school setting provides an opportunity to reach a large proportion of young people [44].

### Study design and participants

**Fig. 1:**
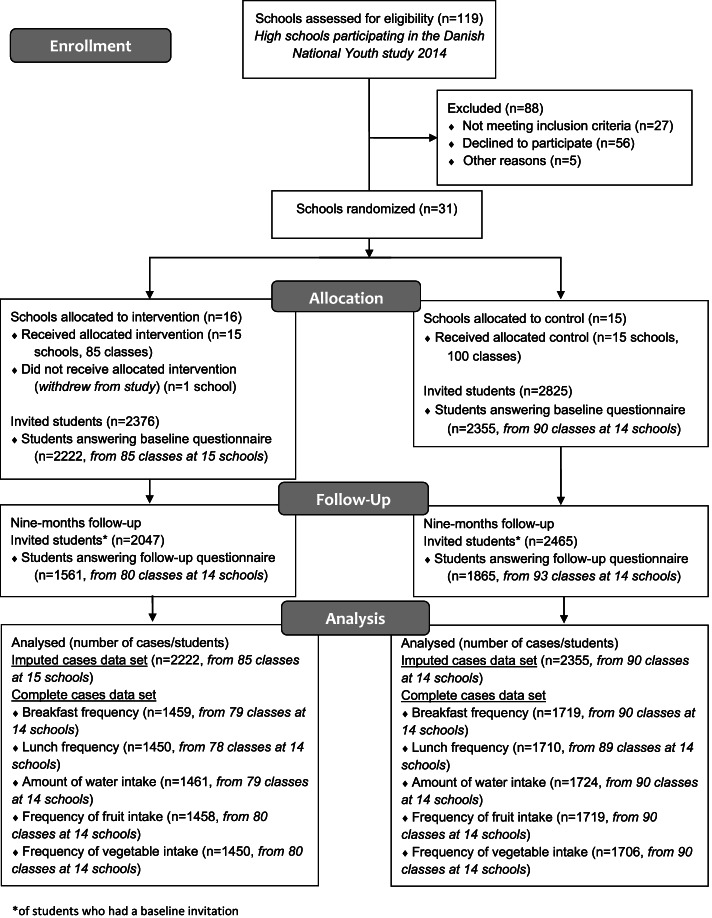
Flow diagram

We invited schools that had previously participated in the Danish National Youth Study 2014 (the sampling strategy has been described in detail elsewhere [40]). Thirty-one of 92 invited schools agreed to participate (33.7%). The participating schools were randomized into the intervention (*n* = 16) and the control group (*n* = 15) by computer-based random number generation. One intervention school withdrew from the study after randomization, leaving a total of 30 schools (Fig. 1) [40]. Sample size calculations showed that a minimum of 26 schools of 224 students in each group were required to detect a between group difference for the primary outcome well-being [40] which make the final school and student sample illustrated in Fig. 1 sufficient. Control schools received no intervention and were asked to continue operating as usual. A refined version of the intervention material was not offered to the control schools before the summer 2020 to avoid that collection of process and effect evaluation data was influenced by spill over effects to control schools.

### Data collection

At the beginning of the school year (August 2016), before intervention start, and at the end of the school year (May 2017) each school was responsible for allocating a 45-min-lesson for students to completed a self-administered online questionnaire (220 items at baseline, 253 items at follow-up) in class after a standardized instruction given by their teacher [40].

Similarly, the principals were to answer an online questionnaire at the two time points, regarding school characteristics, organisational issues, and facilities, as well as the nearby environment (e.g. access to supermarkets and restaurants) [40]. The online format enabled the principals to complete the questionnaire when it was convenient for them.

### Outcome measures

Meal frequency was evaluated by the questions: “*How often do you eat the following meals during the week (Monday to Friday)?”* 1) *“Breakfast (more than a glass of juice or milk)”* and 2) “*Lunch*” with the response options “*no days*”, “*one day*”, “*two days*”, “*three*”, “*four days*” and “*five days*”; And the question “*How often do you eat the following meals on weekends?”* 1) “*Breakfast (more than a glass of juice or milk)”* 2) “*Lunch*” with the response options “*no days*”, “*1 day*” and “*2 days*”. For each meal (breakfast and lunch), the student responses were combined and dichotomized into daily vs 6 days or less a week.

Amount of daily water intake was measured by the question: “*How many glasses of water do you drink on average a day (just water/sparkling water, not squash, coffee, tea or other fluids that contains water)?*” with the response options “*0 glasses*” adding one glass until the final option “*8 glasses or more*” (one glass was estimated to 250 ml). Student responses were dichotomized into ≥4 glasses (1 l or more) a day vs < 4 glasses a day (less than 1 l). The cut-off point of 1 l per day was based on the Nordic Nutrition Recommendation 2012 (NNR 2012) of drinking 1–1.5 l of water per day, depending on activity level, season, and body composition [45].

Intake of fruit and intake of vegetables was measured by the question “*On a normal week: How often do you eat …*? ” 1) “*… fresh fruit”*; 2) “*… vegetables, raw or cooked (not potatoes)*”, with the response options “*never*”, “*less than 1 day a week*”, “*1 day a week*”, “*2-4 days a week*”, “*5-6 days a week*”, “*every day once a day*” and “*every day twice or several times a day*”. To realistically reflect adherence to dietary recommendations, without knowing the amount eaten, the responses for the intake of fruit and vegetables respectively were dichotomized: twice or several times daily (at least twice a day) vs once a day or less.

### Covariates

Covariates strongly correlated with the outcomes of interest were included to increase the precision of the effect estimates [46]. Gender was measured by the question “*What is your gender*” with the categories “*Boy*” and” *Girl*”. Parental occupational social class (OSC) was measured by the questions “*Does your father/mother have a job?*”, “*If no, why does he/she have no job?*”, “*If yes, please say in what place he/she works (for example hospital, bank, restaurant) and please write down exactly what job he/she does there (for example teacher, bus driver)*”. Based on standardised guidelines the answers were coded into OSC from I (high) to V (low) by the research group [47]. We added an extra category for economically inactive parents who receive unemployment benefits, disability pension or other kinds of transfer income. Based on the highest-ranking parent each student was categorized into four levels of OSC: High (I-II e.g., professionals and managerial positions), middle (III-IV, e.g. technical and administrative staff, skilled workers), low (V, unskilled workers and economically inactive) and unclassifiable.

### Statistical analyses

At baseline 4577 answered the questionnaire (response rate = 88.0%), and at follow-up 3426 answered the questionnaire (response rate = 75.9%). .

Baseline data from students and principals were used to characterize the study population and to compare students and schools from the intervention and control group. The main effect analysis applied an intention-to-treat (ITT) approach with multiple imputations of missing data. The imputation was based on variables from the baseline questionnaire which were expected to be associated with the pre-planned outcome measures or loss to follow-up e.g. gender, socio-economic position, and other health behaviours [48].

To allow for correlation between students from the same class and high school, we used multilevel logistic regression analyses to estimate the association between the intervention and the outcomes, with students (level 1) nested within classes (level 2), and classes nested within high schools (level 3). The crude model included the intervention group only. The main model included the intervention group and covariates; baseline level of the specific outcome, gender, and OCS at student-level. School and school class were included as random effects. Intervention group and covariates were included as fixed effects. The intervention was designed as a universal intervention to affect all students. However, as both gender and socioeconomic differences in the outcomes of interest have been demonstrated, we investigated differential effects of the intervention by gender and OSC in explorative subgroup analyses, using the imputed data set.

The statistical analyses were carried out using SAS, version 9.4 (SAS Institute, Inc., Cary, NC) and a significance level of 0.05 was chosen a priori. All analyses were pre-specified in a statistical analysis plan which was approved by all co-authors before the analyses were performed.

### Sensitivity analyses

Sensitivity analyses were carried out on complete case data sets for all outcomes. We also analysed alternative cut-off points for meal frequencies (‘daily Monday-Friday’ vs ‘four days or less’), the amount of daily water intake (‘≥6 glasses a day (1.5 litres)’) and frequency of fruit and vegetable intake (‘at least five days a week’), to investigate whether the intervention only affected students’ intake during school days and higher consumption of water. We also analysed the intervention effect on the combined intake of both fruit and vegetables as they are treated as one food group in dietary recommendations [6].

### Attrition analyses

We performed attrition analyses for all covariates and outcomes of interest on each complete case data set. We performed chi-square test and logistic regression analyses to test for any significant differences between the intervention and control group among students who were either lost to follow-up or had missing data on items included in the complete data sets.

## Results

### Baseline characteristics

At baseline, students at intervention and control schools shared similar characteristics (Table 2). Just above 60% of the students were girls, the mean age was 16 years, and almost half of the students were categorized as high OSC. Half of the students reported to eat breakfast and lunch daily. Larger proportions of students had breakfast daily during weekends compared to school days (Monday-Friday), while smaller proportions had lunch daily during the weekend. Around 70% of students reported drinking at least 1 l of water per day. Less than one-sixth ate fresh fruit and vegetables at least twice a day.

**Table 2:** Baseline characteristics of students participating in the Healthy High School study by intervention group. Values are percentages (numbers) unless stated otherwise

	Imputed cases^**a**^ (*N* = 4755)	
Characteristics of students	Intervention (*n* = 2222)	Control (*n* = 2355)
Girls	61.8 (1372)	64.1 (1541)
Age (years), mean (SD)	16.2 (0.9)	16.3 (1.2)
*Parental Occupational Social Class*		
High social class (I + II)	47.6 (1057)	47.0 (1106)
Middle social class (III + IV)	34.0 (755)	34.0 (801)
Low social class (V)	12.9 (286)	12.9 (303)
Unclassifiable^b^	5.6 (124)	6.2 (145)
*Breakfast frequency*		
Daily intake Monday-Sunday	54.6 (1214)	54.0 (1272)
Daily intake Monday-Friday	67.9 (1508)	66.4 (1563)
Daily intake Saturday-Sunday	74.3 (1650)	75.9 (1787)
*Lunch frequency*		
Daily intake Monday-Sunday	49.9 (1109)	51.7 (1218)
Daily intake Monday-Friday	74.2 (1649)	76.3 (1796)
Daily intake Saturday-Sunday	63.5 (1411)	62.2 (1464)
Daily intake of minimum 1 l^c^ of water	69.8 (1550)	67.8 (1597)
Intake of fresh fruit at least twice a day	15.9 (353)	13.9 (328)
Intake of vegetables at least twice a day	15.5 (344)	14.3 (336)

^a^ Values are presented for imputed data set 1

^b^ Parents who are working, but with too vague information to be categorized into social class I to V

^c^ ≥ 4 glasses (one glass was estimated to contain 250 ml)

Principal questionnaire data showed that more intervention schools had access to fast food (*n* = 12) and healthy lunch (*n* = 12) from nearby restaurants than control schools (fast food *n* = 9, healthy lunch *n* = 6). All schools except one control school had a canteen. Most schools had access to free, cold, clean water from a hygienic source for students, but more intervention schools had water fountains available compared to control schools (data not shown).

### Attrition analyses

Of the 5201 students invited at baseline (Fig. 1), 689 students were lost to followup as they dropped out of school/moved to another school. There were around 30% students with missing data on items included in the complete data sets; breakfast frequency (*n* = 1334, out of total 4512), lunch frequency (*n* = 1352, out of total 4512), amount of daily water intake (*n* = 1327, out of total 4512), frequency of fresh fruit intake (*n* = 1335, out of total 4512), frequency of vegetables intake (*n* = 1356, out of total 4512).

There were no noticeable differences in baseline characteristics between the complete case and imputed data set, except for a higher proportion (almost 10%-points) of students who reported having breakfast in the complete case data set, and a slight tendency of more favourable proportions for the other outcomes compared to the imputed cases data set (Table S1).

We found no significant differences in the attrition between the intervention and the control group according to gender, OSC, baseline breakfast or lunch frequency, amount of daily water intake, and frequency of vegetable intake. However, more students at intervention schools who reported to eat fruit at least twice a day at baseline were lost to follow-up, compared to students at control schools (16.2% versus 12.4%) (data not shown).

### Effect of the HHS intervention on meal frequency and eating habits

In both groups, the proportion of students who reported to have breakfast daily decreased from approximately 50% at baseline to around 40% at follow-up. Around half of students in both groups had lunch daily at baseline, while this proportion was 46.3% for students at intervention schools and 48.6% for students at control schools at follow-up. In both groups, the proportion of students who had four or more glasses (≥1 l) of water a day were approximately 70% at both time points. In both groups 14–15% of the students ate fresh fruit at least twice a day at both baseline and follow-up. Around 15.5% of the intervention students and 14.3% of the control students reported eating vegetables at least twice a day at baseline and this proportion increased to 16% at follow-up in both groups.. Data showed tendencies of gender and socioeconomic differences in all outcomes. A larger proportion of boys reported to eat breakfast and lunch and to drink at least 1 litre of water daily than girls, whereas a larger proportion of girls reported to eat fresh fruit or vegetables, than boys (Table S3 and S4). There was a social gradient in all outcomes. Students of high OSC more frequently reported to have regular meals, drink water and eat fruit and vegetables at least twice a day (Table S3 and S4).

**Fig. 2:**
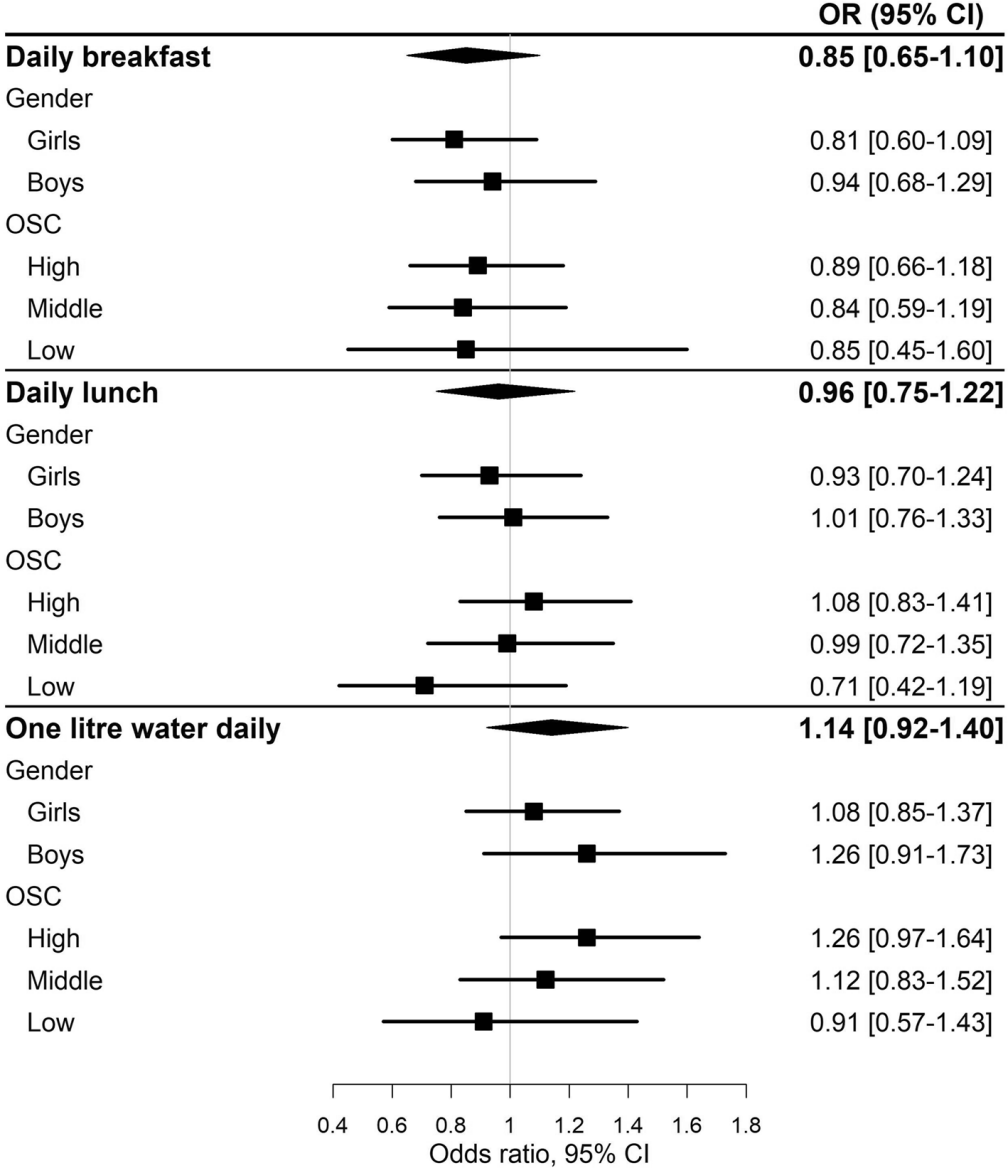
Effect of the Healthy High School intervention at 9-month follow-up on meal frequency and water consumption stratified by gender and parental occupational social class (OSC). Analyses on imputed data sets. Analyses on gender effects were adjusted for baseline level of outcome and OSC. Analyses on OSC effects were adjusted for baseline level of outcome and gender

We found no significant between group difference on frequency of breakfast (OR = 0.85, 95%CI: 0.65;1.10), and lunch intake (OR = 0.96, 95%CI: 0.75;1.22), daily amount of water intake (OR = 1.14, 95% CI: 0.92;1.40), intake of fresh fruit (OR = 1.07, 95% CI: 0.84;1.37) or vegetables (OR = 1.01, 95% CI: 0.77;1.33) in the adjusted ITT analyses (Table 3). Similarly, the sensitivity analyses of complete cases (Table 3) and alternative cut-points (Table S2) did not either show any evidence of an intervention effect. For the daily amount of water intake (< 1,5 l per day), the complete case analysis became borderline significant (OR = 1.26 (95% CI: 1.01;1.57) (Table S2). We found no significant between group difference on any of the outcomes in the explorative subgroup analyses (Fig. 2 and Table S4).

**Table 3:** Effect of the Healthy High School intervention on meal frequency and eating habits at 9-month follow-up. Crude and adjusted analyses of the imputed data sets, and complete case data sets

	Imputed data sets *N* = 40*4577			Complete case data sets		
	% at follow-up	*Primary analysis* Adjusted^a^ OR (95% CI)	Crude OR (95% CI)	% at follow-up	Adjusted^a^ OR (95% CI)	Crude OR (95% CI)
Daily intake of breakfast *(Monday-Sunday)*					*N* = 3178	
Intervention	39.9	0.85 (0.65;1.10)	0.84 (0.60;1.16)	49.8	1.00 (0.82;1.23)	0.97 (0.75;1.25)
Control	42.1	1	1	49.3	1	1
Daily intake of lunch *(Monday-Sunday)*					*N* = 3160	
Intervention	46.3	0.96 (0.75;1.22)	0.91 (0.73;1.15)	54.7	1.05 (0.87;1.26)	1.02 (0.86;1.21)
Control	48.6	1	1	54.2	1	1
Daily intake of minimum 1 litre^b^ of water					*N* = 3185	
Intervention	71.8	1.14 (0.92;1.40)	1.16 (0.93;1.45)	72.5	1.12 (0.90;1.40)	1.18 (0.93;1.50)
Control	68.3	1	1	69.1	1	1
Intake of fresh fruit at least twice a day^c^					*N* = 3177	
Intervention	15.0	1.07 (0.84;1.37)	1.12 (0.87;1.45)	18.2	1.18 (0.93;1.49)	1.17 (0.91;1.52)
Control	14.6	1	1	15.8	1	1
Intake of vegetables at least twice a day^c^					*N* = 3156	
Intervention	16.2	1.01 (0.77;1.33)	1.06 (0.78;1.43)	19.4	1.07 (0.80;1.43)	1.10 (0.79;1.53)
Control	16.0	1	1	17.8	1	1

^a^ Analyses were adjusted for baseline level of outcome, gender and parental occupational social class

^b^ ≥ 4 glasses (one glass was estimated to contain 250 ml)

^c^ Compared to once a day or less

## Discussion

The HHS study is one of the first studies aimed at promoting meal frequency and healthy eating habits among young people in high school. We found no evidence of an effect of the HHS intervention on meal frequency, daily amount of water intake, or intake of fruit and vegetables in the total study sample nor in specific subgroups The intervention may be ineffective due to low implementation levels (implementation failure), misspecification of the working mechanisms (theory failure), and/or measurement error.

### Implementation failure

The HHS intervention was designed to promote regular meals and healthy eating habits by influencing individual, social, and environmental determinants. The canteen initiative addressed environmental determinants i.e., availability, whereas individual determinants such as knowledge and skills were primarily targeted by the HHS app and the HHS curriculum, which both were subject to low implementation. The delivery of the app to the students was delayed significantly due to re-organisations in the app development company and the intended synergy from launching all intervention components at the same time on awareness of the app was compromised (Table 1). This challenged students’ uptake of the app, together with the need for using a code to install the app (Table 1). Raw data from the HHS app showed that 1080 intervention students (45.5% of those invited) downloaded the app, however only 246 (15.7%) students answering the follow-up questionnaire reported to have used the HHS app. Among those who had not used the HHS app the main reason was that they had never heard about the app (27.2%) (unpublished data).

A new educational reform and spending cuts challenged teachers’ implementation of the HHS curriculum. Among 108 teachers responding a process evaluation questionnaire at follow-up, only 39.7% delivered the HHS curriculum, ranging from 0% at some schools to 66.7% at other schools [49]. Between one-fourth and half of intervention students reported to have received each of the five lessons aimed at healthy eating (unpublished data). Thus, important individual determinants were not addressed as planned whereas all except one of the intervention schools implemented the canteen initiative and thereby addressed school-environmental determinants. The process evaluation studies showed that most canteens had followed the consultants’ advice and had changed one or a few products to a healthier alternative e.g. by reducing the content of salt or substituting white bread and pasta with wholegrain products [50]. An intervention at vocational schools used a similar strategy on gradually making a healthier assortment in canteens [51]. Canteens increased their share of healthier food products from 60 to 80% of the assortment over 10 months and increased their sale of healthy products [51]. This effect was found to be even higher when the changes were gradually implemented (60% to 70% to 80%) instead of abruptly (60% to 80%) [51]. Similarly, the interviewed canteen staff in our study underlined the importance of slow and small changes to lure the students in and not ‘scare’ them off [50].

Implementation of structural initiatives [52–54] and complex interventions [55] takes time and 9-month follow-up may be too early to detect the effect of the canteen initiative. However, our process evaluation showed that the canteen staff’s motivation to implement dropped during the intervention year [50] which speaks against this hypothesis.

Looking strictly at breakfast frequency, previous studies have shown that free breakfast at school [56] and low-cost grab and go breakfast at the school [57] is positively associated with breakfast consumption. The HHS intervention did focus on making especially breakfast more available at school, by encouraging canteen staff to provide affordable and healthy breakfast options, or for the school to implement common breakfast for all first-year students. However, the common breakfast was not a mandatory initiative and no schools implemented it. Furthermore, some canteen owners were reluctant to expand their selection of breakfast options, as they did not experience a demand for it.

### The program theory revisited

The program theory assumed that the canteen initiative would prompt the students to buy food in the canteen. However, approximately half of the intervention students never or less than once a week bought food in the canteen for lunch. This proportion remained rather stable towards follow-up. At both baseline and follow-up, more than 60% of students ate packed lunch on most school days (at least 3 days a week). These trends were similar at control schools (unpublished data). Similarly, a Danish study found that 43% of 15–19 year-olds eat food that is prepared at home for lunch e.g. packed lunch [39]. The project group took this into account by sharing recipes for healthy packed lunch, for student to prepare at home, in the app. However, as mentioned previously, few students used the app. Of those who had downloaded the app just 7% had read one or more articles about diet (which included the recipes), 6% used the tracking of water intake, and 5% completed a test on eating and meal habits. In both groups, one-fifth of students at baseline and one-fourth at follow-up bought lunch outside of school (e.g., kiosk, supermarket, fast food restaurants) 1–2 days a week (unpublished data) and many opportunities were available for buying foods and snacks around the schools participating in the HHS study. A previous study found that only 1% of revenues in the canteens at four vocational schools in the Netherlands came from sales of fruit and vegetables [51], which may explain the lack of effect of the HHS intervention on the intake of fruit and vegetables.

The HHS intervention might have benefitted from including a parental component addressing the home environment and parental behaviour as parental involvement is crucial for the promotion of healthy eating habits among 10–18 years-olds [32, 58]. However, based on the needs assessment and recommendations from school staff and students, a parental component was not considered feasible within the high school setting nor beneficial for the students’ uptake of the intervention as they strive for independence. Future qualitative studies should explore parental perspectives on, if, and how they would to be involved in high school-based interventions. Even with a parental component, scholars argue, that school-based interventions often fail, as student behaviour is affected by many other settings than school and families. This calls for more upstream initiatives and a whole system approach to improve health and health-related behaviour in children and young people [59–61].

### Mismatch between intervention focus and outcome measures

Based on previous effective interventions [56, 57, 62, 63], the canteen initiative was developed to increase access to cheap and healthy breakfast, lunch and snacks at the school. Meanwhile, the initiative ended up focusing more on the nutritional values of the food than intended as the recruited intervention provider - the canteen consultant - used the scoring system “*Kantinetjekket|Buffet*” (in English “*Canteencheck|Buffet*”) (Table 1) . Changes in nutritional values e.g., salt content were not captured by our broad outcomes of meal frequency and eating habits. We had to keep the number of items measuring food intake to a minimum due to the many outcomes of interests of the overall HHS study. As we only measured meal frequency and overall eating habits, we do not know whether the intervention resulted in e.g., a healthier breakfast among students eating breakfast. However, as many of the canteen changes were quite invisible e.g., adding more wholegrain flour in the bread, the students would probably not have noticed and been able to report that they ate more wholegrain when they bought lunch in the school canteen. A recent review found that school-based interventions aimed at improving food behaviours through nudging are positively associated with food selection, but often fail to detect an effect on actual consumption as it is not measured, or measures on food waste is not taken into consideration [64].

### Strengths and limitations

The previous sections have elaborated on study limitations related to implementation levels, programme theory, and outcome measures. Strengths of the HHS study include; 1) the RCT design, 2) the high response rate of students, 3) the use of multilevel modelling taking the clustering of the data into account, 4) the long duration of the intervention, 5) the systematic, transparent planning process guided by the Intervention Mapping Protocol [41] (Bonnesen et al. *unpublished*), based on a thorough mixed-methods needs assessment analysis (Bonnesen et al. *unpublished*), use of behavioural and environmental change theory [65] and the best available evidence in the field [30, 32]. Moreover, the HHS intervention design acknowledges the complex drivers of young peoples’ health behaviour including the clustering of behaviour and uses several initiatives to target determinants on both an environmental, interpersonal, and individual level.

### Public health implications

Scholars have called for environmental initiatives [55, 66–69], and the HHS canteen initiative could inspire future interventions. The process evaluation showed promising results for the initiative in changing the canteen selection. Canteen staff expressed appreciation for the consultant‘s tailored advice and the “health-check” of the canteen [50]. However, their motivation lowered during the intervention period. Future studies may consider multiple consulting meetings to uphold the motivation and sustain positive changes in the school canteen. Environmental initiatives such as provision of free school meals which has shown positive health effects in younger children in Sweden [70] could be interesting to test in this age group.

We found a relative low use of the app, which has been argued could be due to implementation failure. Moreover, some students reported that reasons for not using the app were that the app was not interesting that they had other apps already with similar features. This study thereby highlights the possible pitfalls and barriers to the use of an app. However, we still find it relevant to explore how technology as apps can be used to target important individual determinants in interventions.

The HHS intervention aimed to support students in replacing unhealthy choices with more healthy choices. Future studies should investigate the effect of the intervention on intake of unhealthy snacks and SSB and the effect of the initiative on all outcomes of interests at 20-month follow-up to explore the sustainability of the intervention and the hypothesis that implementation takes time [40]. Furthermore the interplay between snack behaviour and meal frequencies is an interesting area of research as some might skip meals for snacks or report meals as snacks. Moreover, it is warranted to develop valid outcome measures to detect the possible small changes in student’s eating habits that might have occurred from the changes we observed in the canteens.

Despite showing no evidence of an effect of the HHS intervention, the descriptive findings underline the continued need for effective initiatives targeting meal frequency and eating habits among high school students in Denmark, to ensure health and well-being in both youth and adult life. Future studies could benefit from looking beyond the school setting and include several systems [59–61].

## Conclusion

This study evaluated the effect of the multicomponent school-based HHS intervention in 16-year-old students on meal frequencies, amount of daily water intake, intake of fresh fruit and intake of vegetables at 9-month follow-up. We found no evidence of an effect of the intervention on any of the outcomes in the total study population nor specific subgroups.

The descriptive data shows that at least 40% of Danish high school students skip breakfast and/or lunch, and approximately 70% do not drink enough water. Also, only one out of seven eats fresh fruit or vegetables at least twice a day, respectively. This highlights the continued need for effective interventions targeting meal frequencies and eating habits among young people in Denmark. Based on some of the newest literature it is warranted that future interventions should include the whole system of young people’s lives and focus more on interventions at several structural levels, and not just the school setting. Moreover, is it needed to explore further how to successfully engage high schools in health promoting activities.

## Supplementary Information

**Additional file 1.**

**Additional file 2.**

**Additional file 3.**

**Additional file 4.**

## Data Availability

The data sets generated and/or analysed during the current study are not publicly available due to the sensitivity of data but are available from the corresponding author on reasonable request.

## Abbreviations

SSB: Sugar-sweetened beverage;
HHS: Healthy High School study
app: Smartphone application
OSC: Parental occupational social class
ITT: Intention-to-treat
RCT: Randomized controlled study
