# Supplementary material for: Effect of the multicomponent healthy high school intervention on meal frequency and eating habits among high school students in Denmark: a cluster randomized controlled trial
Source: Int J Behav Nutr Phys Act. 2022 Feb 4;19:12. doi: 10.1186/s12966-021-01228-2 (PMC8815150; doi:10.1186/s12966-021-01228-2)
Supplement: Supplementary file 1 — Additional file 1. [file 12966_2021_1228_MOESM1_ESM.docx]

| **Table S1** Baseline characteristics of students participating in the Healthy High School study based on each of the complete case data sets; Breakfast frequency, Lunch frequency, Water consumption, Frequency of fresh fruit and Frequency of vegetables. Values are percentages (numbers) unless stated otherwise. I=Intervention, C=Control. | | | | | | | | | | |
| --- | --- | --- | --- | --- | --- | --- | --- | --- | --- | --- |
|  | Breakfast frequency  N=3178 | | Lunch frequency  N=3160 | | Water consumption  N=3185 | | Frequency of fresh fruit  N=3177 | | Frequency of vegetables  N=3156 | |
| **Characteristics of students** | I  (n=1459) | C  (n=1719) | I  (n=1450) | C  (n=1710) | I  (n=1460) | C  (n=1724) | I  (n=1458) | C  (n=1719) | I  (n=1450) | C  (n=1706) |
| Girls | 63.5 (927) | 66.4 (1142) | 63.5 (921) | 66.4 (1136) | 63.5 (927) | 66.4 (1145) | 63.6 (927) | 66.6 (1144) | 63.6 (922) | 66.7 (1138) |
| Age (years), mean (SD) | 16.2 (0.7) | 16.2 (1.1) | 16.2 (0.7) | 16.2 (1.1) | 16.2 (0.7) | 16.2 (1.1) | 16.2 (0.7) | 16.2 (1.1) | 16.2 (0.7) | 16.2 (1.1) |
| Parental Occupational Social Class |  |  |  |  |  |  |  |  |  |  |
| High social class (I+II) | 50.0 (730) | 49.0 (842) | 50.0 (725) | 49.0 (837) | 50.1 (732) | 48.9 (843) | 50.0 (729) | 49.0 (842) | 50.1 (727) | 48.9 (834) |
| Middle social class (III+IV) | 35.4 (517) | 33.1 (569) | 35.6 (516) | 33.2 (567) | 35.3 (516) | 33.2 (573) | 35.5 (517) | 33.3 (572) | 35.5 (515) | 33.4 (570) |
| Low social class (V) | 10.4 (152) | 12.0 (206) | 10.4 (151) | 11.9 (203) | 10.5 (153) | 11.7 (202) | 10.4 (152) | 11.7 (201) | 10.3 (149) | 11.7 (199) |
| Unclassifiable ^a^ | 4.1 (60) | 5.9 (102) | 4.0 (58) | 6.0 (103) | 4.1 (60) | 6.2 (106) | 4.1 (60) | 6.1 (104) | 4.1 (59) | 6.0 (103) |
|  |  |  |  |  |  |  |  |  |  |  |
| Daily intake of breakfast | 58.7 (856) | 58.1 (998) | - | - | - | - | - | - | - | - |
| Daily intake of lunch | - | - | 52.4 (760) | 53.7 (919) | - | - | - | - | - | - |
| Daily intake of minimum 1 litre ^b^ of water | - | - | - | - | 71.0 (1037) | 68.0 (1173) | - | - | - | - |
| Intake of fresh fruit at least twice a day | - | - | - | - | - | - | 15.8 (231) | 14.5 (250) | - | - |
| Intake of vegetables at least twice a day | - | - | - | - | - | - | - | - | 16.3 (236) | 14.6 (249) |
| ^a^ Parents who are working, but for whom the information was too vague for categorizing into social class I to V  ^b^ ≥ 4 glasses (one glass was estimated to contain 250 ml) | | | | | | | | | | |
