# Supplementary material for: Effect of the multicomponent healthy high school intervention on meal frequency and eating habits among high school students in Denmark: a cluster randomized controlled trial
Source: Int J Behav Nutr Phys Act. 2022 Feb 4;19:12. doi: 10.1186/s12966-021-01228-2 (PMC8815150; doi:10.1186/s12966-021-01228-2)
Supplement: Supplementary file 2 — Additional file 2. [file 12966_2021_1228_MOESM2_ESM.docx]

| Table S2 Effect of the Healthy High School intervention at 9-month follow-up. Analyses of the imputed data sets, adjusted (primary analyses) and crude and complete cases, adjusted and crude. | | | | | | |
| --- | --- | --- | --- | --- | --- | --- |
|  | **Imputed data sets**  N=40*4577 | | | **Complete case data set** | | |
|  | % at follow-up | Adjusted^a^  OR (95% CI) | Crude  OR (95% CI) | % at follow-up | Adjusted^a^  OR (95% CI) | Crude  OR (95% CI) |
| Daily intake of breakfast *(Monday-Friday)* |  |  |  | N=3178 | | |
| Intervention | 50.0 | 0.84 (0.64;1.10) | 0.84 (0.58;1.22) | 60.0 | 0.96 (0.78;1.18) | 0.95 (0.71;1.28) |
| Control | 51.5 | 1 | 1 | 59.3 | 1 | 1 |
| Daily intake of lunch *(Monday-Friday)* |  |  |  | N=3160 | | |
| Intervention | 69.6 | 0.95 (0.71;1.27) | 0.88 (0.64;1.22) | 79.2 | 1.09 (0.80;1.49) | 1.01 (0.73;1.41) |
| Control | 71.0 | 1 | 1 | 77.8 | 1 | 1 |
| Daily intake of minimum 1.5 litre^b^ of water |  |  |  | N=3185 | | |
| Intervention | 36.1 | 1.20 (0.99;1.46) | 1.17 (0.95;1.44) | 36.3 | **1.26 (1.01;1.57)** | 1.22 (0.99;1.52) |
| Control | 31.6 | 1 | 1 | 31.9 | 1 | 1 |
| Intake of fresh fruit at least 5 days a week^c^ |  |  |  | N=3177 | | |
| Intervention | 49.2 | 1.02 (0.84;1.22) | 1.04 (0.85;1.26) | 50.0 | 0.98 (0.80;1.21) | 1.01 (0.80;1.28) |
| Control | 48.2 | 1 | 1 | 48.7 | 1 | 1 |
| Intake of vegetables at least 5 days a week^c^ |  |  |  | N=3156 | | |
| Intervention | 59.5 | 1.12 (0.93;1.36) | 1.12 (0.87;1.45) | 62.1 | 1.16 (0.94;1.43) | 1.13 (0.85;1.50) |
| Control | 58.6 | 1 | 1 | 59.8 | 1 | 1 |
| Intake of both fruit and vegetables daily^d^ |  |  |  | N=2380 | | |
| Intervention | 8.8 | 1.05 (0.78;1.42) | 1.10 (0.78;1.54) | 11.2 | 1.20 (0.86;1.69) | 1.25 (0.90;1.74) |
| Control | 8.2 | 1 | 1 | 9.1 | 1 | 1 |
| ^a^ Analyses were adjusted for baseline level of outcome, gender and parental occupational social class  ^b^ ≥ 6 glasses (one glass was estimated to contain 250 ml)  ^c^ Compared to 4 days a week or less  ^d^ At least twice a day for both fruit and vegetables | | | | | | |
