# Supplementary material for: Effect of the multicomponent healthy high school intervention on meal frequency and eating habits among high school students in Denmark: a cluster randomized controlled trial
Source: Int J Behav Nutr Phys Act. 2022 Feb 4;19:12. doi: 10.1186/s12966-021-01228-2 (PMC8815150; doi:10.1186/s12966-021-01228-2)
Supplement: Supplementary file 3 — Additional file 3. [file 12966_2021_1228_MOESM3_ESM.docx]

| Table S3 Effect of the Healthy High School intervention at 9-month follow-up on meal frequency and water consumption stratified by gender and parental occupational social class (OSC). Analyses on imputed data sets. | | | | | | | | | |
| --- | --- | --- | --- | --- | --- | --- | --- | --- | --- |
|  | **Daily intake of breakfast^a^**  N=40*4577 | | | **Daily intake of lunch^a^**  N=40*4577 | | | **Daily intake of minimum**  **1 litre^b^ of water**  N=40*4577 | | |
|  | % at  baseline | % at  follow-up | Adjusted OR  (95% CI) | % at  baseline | % at  follow-up | Adjusted OR  (95% CI) | % at  baseline | % at  follow-up | Adjusted OR  (95% CI) |
| Girls |  |  |  |  |  |  |  |  |  |
| Intervention | 53.4 | 39.3 | 0.81 (0.60;1.09) ^c^ | 48.1 | 43.9 | 0.93 (0.70;1.24) ^c^ | 67.9 | 69.8 | 1.08 (0.85;1.37) ^c^ |
| Control | 52.9 | 43.3 | 1 | 49.1 | 45.8 | 1 | 65.2 | 65.9 | 1 |
| Boys |  |  |  |  |  |  |  |  |  |
| Intervention | 56.7 | 40.8 | 0.94 (0.68;1.29) ^c^ | 52.8 | 50.2 | 1.01 (0.76;1.33) ^c^ | 72.7 | 75.2 | 1.26 (0.91;1.73) ^c^ |
| Control | 56.0 | 40.0 |  | 56.3 | 53.9 | 1 | 72.5 | 72.7 | 1 |
| High OSC |  |  |  |  |  |  |  |  |  |
| Intervention | 59.2 | 44.1 | 0.89 (0.66;1.18) ^d^ | 51.9 | 50.0 | 1.08 (0.83;1.41) ^d^ | 72.0 | 75.0 | 1.26 (0.97;1.64) ^d^ |
| Control | 56.6 | 45.6 | 1 | 51.3 | 48.3 | 1 | 69.8 | 69.8 | 1 |
| Middle OSC |  |  |  |  |  |  |  |  |  |
| Intervention | 53.6 | 39.6 | 0.84 (0.59;1.19) ^d^ | 48.5 | 46.8 | 0.99 (0.72;1.35) ^d, e^ | 69.3 | 71.9 | 1.12 (0.83;1.52) ^d^ |
| Control | 56.2 | 42.7 | 1 | 51.4 | 50.4 | 1 | 67.5 | 69.2 | 1 |
| Low OSC |  |  |  |  |  |  |  |  |  |
| Intervention | 45.8 | 28.3 | 0.85 (0.45;1.60) ^d^ | 50.4 | 36.7 | 0.71 (0.42;1.19) ^d^ | 64.0 | 61.2 | 0.91 (0.57;1.43) ^d^ |
| Control | 42.9 | 32.7 | 1 | 54.5 | 46.5 | 1 | 63.4 | 62.7 | 1 |
| ^a^ Monday-Sunday  ^b^ ≥ 4 glasses (one glass was estimated to contain 250 ml)  ^c^ Analyses were adjusted for baseline level of outcome and parental occupational social class  ^d^ Analyses were adjusted for baseline level of outcome and gender  ^e^ Analysis for imputed data set 39 would not converge and were removed from the analysis, leaving the analysis with a N=39*4755 | | | | | | | | | |
