# Supplementary material for: Effect of the multicomponent healthy high school intervention on meal frequency and eating habits among high school students in Denmark: a cluster randomized controlled trial
Source: Int J Behav Nutr Phys Act. 2022 Feb 4;19:12. doi: 10.1186/s12966-021-01228-2 (PMC8815150; doi:10.1186/s12966-021-01228-2)
Supplement: Supplementary file 4 — Additional file 4. [file 12966_2021_1228_MOESM4_ESM.docx]

| Table S4 Effect of the Healthy High School intervention at 9-month follow-up on daily intake of fruit at least twice a day, vegetables at least twice a day and both fruit and vegetables at least twice a day stratified by gender and parental occupational social class (OSC). Analyses on imputed data sets. | | | | | | | | | |
| --- | --- | --- | --- | --- | --- | --- | --- | --- | --- |
|  | **Daily intake of fruit at least twice a day** N=40*4755 | | | **Daily intake of vegetables at least twice a day**  N=40*4755 | | | **Daily intake of both fruit and vegetables at least twice a day** N=40*4755 | | |
|  | % at  baseline | % at  follow-up | Adjusted OR  (95% CI) | % at  baseline | % at  follow-up | Adjusted OR  (95% CI) | % at  baseline | % at  follow-up | Adjusted OR  (95% CI) |
| Girls |  |  |  |  |  |  |  |  |  |
| Intervention | 18.2 | 17.8 | 1.12 (0.85;1.47) ^a^ | 17.9 | 18.6 | 0.96 (0.69;1.34) ^a^ | 9.5 | 10.1 | 1.03 (0.73;1.44) ^a, c^ |
| Control | 15.9 | 16.0 | 1 | 16.6 | 18.8 | 1 | 7.5 | 9.1 | 1 |
| Boys |  |  |  |  |  |  |  |  |  |
| Intervention | 12.2 | 10.5 | 0.98 (0.66;1.46) ^a^ | 11.5 | 12.4 | 1.14 (0.77;1.70) ^a^ | 6.2 | 6.8 | 1.19 (0.74;1.90) ^a^ |
| Control | 10.4 | 12.1 | 1 | 10.1 | 11.0 | 1 | 5.9 | 6.6 | 1 |
| High OSC |  |  |  |  |  |  |  |  |  |
| Intervention | 18.5 | 16.7 | 1.01 (0.72;1.41) ^b^ | 19.5 | 19.6 | 1.06 (0.76;1.48) ^b^ | 10.4 | 11.0 | 1.12 (0.76;1.65) ^b^ |
| Control | 15.5 | 17.1 | 1 | 17.9 | 19.2 | 1 | 8.7 | 10.1 | 1 |
| Middle OSC |  |  |  |  |  |  |  |  |  |
| Intervention | 14.4 | 15.2 | 1.13 (0.80;1.60) ^b^ | 13.1 | 13.5 | 0.84 (0.58;1.22) ^b^ | 6.2 | 6.8 | 0.90 (0.56;1.44) ^b^ |
| Control | 12.6 | 14.0 | 1 | 12.0 | 15.0 | 1 | 5.7 | 7.5 | 1 |
| Low OSC |  |  |  |  |  |  |  |  |  |
| Intervention | 12.2 | 9.40 | 1.33 (0.68;2.60) ^b^ | 9.4 | 11.5 | 1.55 (0.73;3.28) ^b^ | 7.0 | 7.0 | 1.46 (0.54;3.95) ^b^ |
| Control | 12.9 | 7.9 | 1 | 9.6 | 9.2 | 1 | 3.6 | 4.0 | 1 |
| ^a^ Analyses were adjusted for baseline level of outcome and parental occupational social class  ^b^ Analyses were adjusted for baseline level of outcome and gender  ^c^ Analysis for imputed data set 6 would not converge and were removed from the analysis, leaving the analysis with a N=39*4755 | | | | | | | | | |
